# Large differences in variability for genes associated with antimalarial drug resistance between samples from Tanzania and Ethiopia

**DOI:** 10.1186/1475-2875-13-S1-P87

**Published:** 2014-09-22

**Authors:** Lemu Golassa, Nizar Enweji, Göte Swedberg, Erasmus Kamugisha, Angel Choy

**Affiliations:** 1 Uppsala University, Uppsala, Sweden; 2 Catholic University of Health and Allied Sciences, Mwanza, Tanzania

## Background

Changes in antimalarial drug policy lead to selection of the most successful parasite clones. In spite of a change to Artemisinin Combination Therapy (ACT), we have earlier shown a low degree of variability for the chloroquine resistance marker pfcrt in samples of *Plasmodium falciparum* from Ethiopia with 100% carrying the crucial K76T variant [1].

## Materials and methods

The study material was collected in the Shalla district, Oromia, Ethiopia, and in different parts of Tanzania. Genetic polymorphisms in the genes *pfmdr1* and *pfubp1* were analysed by PCR and nucleotide sequence determinations.

## Results

A surprising finding was that a majority of Ethiopian isolates carried the wild type variant of *pfmdr1*. The majority of samples from different regions of Tanzania were wild type for both *pfcrt* and *pfmdr1*. Analysis of a variable linker region in *pfmdr1* showed substantial variation in samples from Tanzania, but minimal variation in samples from Ethiopia. The variable part consists of consecutive NDN residues. The dominating variant in Ethiopians was 7/2/9 and 7/2/10 in Tanzania. The same pattern was seen for a variable part of the *pfubp1* gene, where all Ethiopian samples were identical to 3D7. In 19 Tanzanian samples only 8 were identical to 3D7 with 5 other variants. A polymorphism at codon 1528, detected previously in Kenyan samples [2] was found in 2 Tanzanian samples.

## Conclusions

While the presence of the mutant variant of *pfcrt* in the Ethiopian samples can be explained by continued use of chloroquine in Ethiopia for treatment of *P. vivax*, the selection of wild type *pfmdr1* could be a consequence of using ACT for treatment of *P. falciparum*. In general, the variability in both studied genes was greater in Tanzania than in Ethiopia. There are no data yet to link the variability in *pfubp1* to efficacy of ACT.
